# Editorial: The Role of Microbial Communities in Tropical Ecosystems

**DOI:** 10.3389/fmicb.2016.01805

**Published:** 2016-11-11

**Authors:** Silvia Pajares, Brendan J. M. Bohannan, Valeria Souza

**Affiliations:** ^1^Unidad Académica de Ecología y Biodiversidad Acuática, Instituto de Ciencias del Mar y Limnología, Universidad Nacional Autónoma de MéxicoMexico City, Mexico; ^2^Department of Biology, Institute of Ecology and Evolution, University of OregonEugene, OR, USA; ^3^Departamento de Ecología Evolutiva, Instituto de Ecología, Universidad Nacional Autónoma de MéxicoMexico City, Mexico

**Keywords:** microbial ecology, tropical ecosystem, environmental factors, land use change, microbial processes and diversity

Microorganisms represent the largest proportion of the Earth's biodiversity and play an essential role in ecosystem processes, providing functions that ultimately sustain all of life (Falkowski et al., 2008; Prosser, 2012). Understanding the link between ecosystem functioning and the distribution of microbial diversity is essential to predict ecosystem responses to a changing environment (de Vries and Shade, 2013; Logue et al., 2015). With the rapid development of molecular-based techniques, a new interest in understanding the distribution patterns and functional traits of microbial communities has emerged (Fuhrman, 2009; Strickland et al., 2009; Bradford and Fierer, 2012; Krause et al., 2014). However, most of this research has been focused in temperate regions, and the principal mechanisms controlling microbial community variation within the tropics are poorly known.

Tropical ecosystems are different in important ways from those of temperate regions. They are a major reservoir of plant and animal biodiversity and play important roles in global climate regulation and biogeochemical cycling (Gibson et al., 2011; Townsend et al., 2011). They are also under great threat due to the conversion of tropical ecosystems to other uses (Bawa et al., 2004; Stork et al., 2009). Thus, in the context of global change, it is crucial to understand how environmental factors, biogeographic patterns, and land use changes interact to influence the structure and function of microbial communities in these ecosystems.

It is possible that different rules apply to microbial life in tropical ecosystems. For instance, elevated nitrogen deposition by anthropogenic activities may exacerbate phosphorus deficiency in tropical regions, in ways uncommon in temperate ecosystems (Vitousek et al., 2010). However, it is poorly understood how phosphorus availability affects soil microbes (Heuck et al., 2015), or how microbial processes interact with nitrogen deposition in tropical ecosystems (Hietz et al., 2011). Moreover, the distribution of microbes not only is related to environmental factors, but also can vary in relation to temporal and spatial scale (Martiny et al., 2006; Shade et al., 2012). These factors influence biodiversity patterns of larger organisms, but their role in microbial diversity remains unclear (Barberán et al., 2014), especially in tropical systems. In addition, little is known about microbial community responses to disturbance in the tropics. Land use is one of the main drivers of biodiversity alteration in plant and animal communities, especially in tropical areas, where natural ecosystems such as forests are being rapidly altered by conversion to agriculture and other uses (Barnes et al., 2014). Understanding the effects of land use change on soil microorganisms is also a major conservation frontier.

The primary aim of this Research Topic is to explore the environmental and anthropological factors controlling the composition and functional diversity of microbial communities in tropical systems. For example, Fanin et al. reported that temporal differences in nitrogen and phosphorus availability in tropical forest soils are critical for the activity and the structure of microbial decomposer communities. Tripathi et al. observed that within tropical rainforests there is a strong degree of ecological differentiation in soil and, as a consequence soil properties together with bacterial and fungal communities varied significantly between forest types. Lee et al. documented the distribution of methanotrophs and methanogens along a depth gradient in a tropical rice paddy, and looked for patterns with oxygen, methane, and organic carbon concentrations in these soils. The influence of environmental and anthropogenic factors, such as nitrogen deposition and land-use change, on the key players in the nitrogen cycle was also assessed in a general review Pajares and Bohannan. This review highlights the large gaps in our understanding of microbially mediated nitrogen processes in tropical forest soils and identifies important areas for future research.

Due to ongoing and widespread deforestation of tropical ecosystems for pasture and crop cultivation, there is an urgent need to evaluate their soil biological diversity. This Research Topic presents new findings on the impact of rainforest conversion on soil microbial community structure, diversity and ultimately functional traits Schneider et al. Particularly in the wet season, land use change from rainforest to agriculture reduced the abundance of different functional microbial groups related to the soil carbon and nitrogen cycles Lammel et al. There is limited knowledge regarding the diversity of important groups of microorganisms in the tropics. Thus, other contributions studied how abundant lineages in soils, such as Acidobacteria Navarrete et al., and Verrucomicrobia Ranjan et al., responded to forest-to-pasture conversion. These studies demonstrate that such groups can be useful as indicators of agricultural impact on tropical ecosystems. Furthermore, the isolation and characterization of two novel moderately thermophilic and acid-tolerant obligate methanotrophs recovered from a tropical methane seep topsoil habitat were described Islam et al.

Some contributions to this Research Topic have focused on how land management practices impact microbially-mediated processes in tropical soils through the alteration of microbial communities. Reverchon et al. showed the influence of different tree plantation systems on nitrogen retention and the abundance of nitrogen functional genes, while Wood et al. concluded that the direct effect of farm management (rather than the indirect effect through changes in the taxonomic and functional diversity of microbial communities) was the dominant control of nutrient loss from smallholder tropical agriculture. Both studies emphasize the necessity of further research on incorporating microbial dynamics into plantation management to improve productivity while mitigating soil fertility loss. Xiong et al. observed that sustainable agricultural management regimes, such as crop rotation, of commercially important crops in tropical regions significantly increased soil fungal diversity and might be a meaningful strategy to prevent vanilla *Fusarium* wilt disease.

Finally, one of the biggest threats facing frog populations in montane tropical systems is a lethal skin disease caused by a chytrid fungus. The composition of the frog skin microbiota can strongly influence many facets of frog health and disease resistance. Field survey data published as part of our Research Topic has demonstrated that the frog skin microbiota is producing broadly similar sets of anti-fungal metabolites across different host species and sites Belden et al.

In summary, the contributions to this Research Topic showcase the current knowledge regarding microbial ecology in tropical ecosystems, identify many challenges and questions that remain to be addressed and open up new horizons in our understanding of the environmental and anthropological factors controlling microbial communities in these important ecosystems.

## Author contributions

SP produced the first draft of the editorial, and all authors edited the editorial.

### Conflict of interest statement

The authors declare that the research was conducted in the absence of any commercial or financial relationships that could be construed as a potential conflict of interest.
